# Supplementary material for: MetaQTL: a package of new computational methods for the meta-analysis of QTL mapping experiments
Source: BMC Bioinformatics. 2007 Feb 8;8:49. doi: 10.1186/1471-2105-8-49 (PMC1808479; doi:10.1186/1471-2105-8-49)
Supplement: Additional File 7 — MetaQTL Package : jar file and tutorial. This Zip archive contains both the MetaQTL JAR file and the files of the tutorial. [file 1471-2105-8-49-S7.zip › org.inra.metaqtl/doc/org/metaqtl/package-frame.html]

org.metaqtl


org.metaqtl

Interfaces 
  
*IBioGenomeProperties*
  
*IBioLGroupProperties*
  
*IBioLocusProperties*
  
*IMetaAlgorithm*
  
*IMetaQtlConstants*

Classes 
  
ChromCluster
  
Chromosome
  
CMarkerSequence
  
EMCriteria
  
EMResult
  
MapCluster
  
MetaChrom
  
MetaDico
  
MetaMap
  
MetaQtlAnalysis
  
MetaQtlData
  
MetaQtlModel
  
MetaQtlResult
  
MultiFitResult
  
Qtl
  
QtlPartition
  
Tree
  
TreeNode
